# Diagnostic Value of ^11^C-PIB PET/MR in Cardiac Amyloidosis

**DOI:** 10.3389/fcvm.2022.830572

**Published:** 2022-03-16

**Authors:** Xiao Bi, Baixuan Xu, Jiajin Liu, Guanyun Wang, Jing An, Xiaojun Zhang, Ruimin Wang, Wei Dong, Zhiwei Guan

**Affiliations:** ^1^Department of Nuclear Medicine, The First Medical Centre, Chinese PLA General Hospital, Beijing, China; ^2^Department of Cardiology, The Sixth Medical Centre, Chinese PLA General Hospital, Beijing, China; ^3^Siemens Healthcare Ltd., Guangdong, China; ^4^National Clinical Research Center for Geriatric Diseases, Chinese PLA General Hospital, Beijing, China

**Keywords:** ^11^C-PIB, PET/MR, cardiac amyloidosis, non-invasive diagnosis, LGE, ECV, TBR

## Abstract

**Background:**

The thioflavin T derivative, ^11^C-Pittsburgh-B (PIB), is used for Alzheimer's disease imaging because it specifically binds to β-amyloid protein deposits in the brain. The aim of this study was to estimate the diagnostic value of combined ^11^C-PIB positron emission tomography/magnetic resonance (PET/MR) in cardiac amyloidosis (CA).

**Methods:**

We enrolled 23 heart failure patients with suspected CA based on echocardiographic and electrocardiograph findings. All patients underwent cardiac ^11^C-PIB PET/MR and non-cardiac biopsy within one week. We also enrolled eight healthy volunteers that underwent cardiac ^11^C-PIB PET/MR as a control group. The cardiac magnetic resonance (CMR) protocol included cine imaging, late gadolinium enhancement (LGE), and native and post-contrast T1 mapping. Extracellular volume (ECV) was measured using pre- and post-contrast T1 mapping images. LVEF, IVSD, LVPW, LVmass, LVESV, LVEDV, native T1 value, ECV, and maximum uptake of myocardial tissue-to-blood background ratio (TBR) values were obtained from PET/MR images in all patients and healthy subjects.

**Results:**

Thirteen out of twenty-three heart failure patients were clinically diagnosed with CA. The remaining 10 patients were CA-negative (non-CA patient group). Twelve of the thirteen CA patients showed diffuse transmural LGE patterns, whereas LGE was either absent or patchy in the non-CA patients. The diagnostic sensitivity and specificity of TBRmax were 92.3 and 100%, respectively, at a cut-off value of 1.09. Several CMR imaging parameters (LVEF, IVSD, LVmass, LVEDV, LVESV, LVPW, native T1 value and ECV) and TBR showed significant differences between CA patients, non-CA patients, and healthy controls (*P* < 0.05). Native T1 mapping values positively correlated with TBRmax values in CA and non-CA patients (*r* = 0.38, *P* = 0.0004).

**Conclusions:**

^11^C-PIB PET/MRI is a valuable tool for the accurate and non-invasive diagnosis of CA because it distinguishes CA patients from non-CA patients and healthy subjects with high specificity and sensitivity. Moreover, native T1 mapping values positively correlated with TBRmax values in CA and non-CA patients. In the future, larger cohort studies are necessary to confirm our findings.

## Introduction

Amyloidosis refers to a group of systemic diseases caused by extracellular and/or intracellular accumulation of insoluble misfolded amyloid protein fibrils, which progressively damage the structure and function of related organs (1). Cardiac amyloidosis (CA) is a type of restrictive cardiomyopathy caused by the accumulation of misfolded amyloid protein deposits in the myocardium (2). Heart failure is the main cause of death and morbidity in CA patients, which manifests either as a primary disease or as part of systemic amyloidosis (3). Endocardial biopsy (EB) is the current gold standard for the clinical diagnosis of myocardial amyloidosis (4). However, EB is an invasive procedure that cannot be performed routinely. EB is also associated with high false-negative biopsy interpretation rates. Moreover, it does not provide sufficient clinical information regarding the status of the disease and is not effective for determining prognosis or response to treatment.

Cardiovascular magnetic resonance (CMR) imaging with late gadolinium enhancement (LGE) is the most commonly performed non-invasive technique for characterizing myocardial tissue abnormalities in a wide spectrum of cardiomyopathies (5). Although multiple LGE distributions have been described for cardiac amyloidosis, sub-endocardial and transmural LGE patterns are most commonly observed in cardiac amyloidosis and serve as diagnostic markers (6). However, LGE-CMR is not amenable for the early recognition of mild myocardial amyloidosis during differential diagnosis (7, 8). LGE-CMR is also not suitable for suspected CA patients with severe renal impairment (9, 10). Non-contrast T1 mapping is performed before administering contrast agents to quantify the direct signal from the myocardium (11). Several studies have shown that native T1 values are slightly elevated in focal and diffuse fibrosis (12, 13), edema, and inflammation (14). Boomen et al. reported that myocardial T1 values were significantly higher for patients with amyloidosis, including those without any confirmed cardiac involvement through biopsy or decreased cardiac function (15). However, a major disadvantage of native T1 mapping is that the results can vary significantly based on the type of scanners and magnetic field intensities (1.5T vs. 3T) used for the analysis. ECV (extracellular volume) is another early marker of cardiac involvement in patients with amyloidosis (confirmed by biopsy) and is more reproducible than absolute T1 values (16). However, in the absence of biopsy confirmation, ECV values may overlap with other cardiomyopathy pathologies and limit the specificity of ECV in the early detection of amyloidosis (8).

The thioflavin-T derivate, ^11^C-Pittsburgh B (PIB), is used for the diagnostic imaging of patients with Alzheimer's disease because it binds with high-affinity to fibrillar β-amyloid protein deposits in the brain (17). Amyloid positron emission tomography (PET) imaging can be used for quantitative analysis of cardiac amyloidosis because it shows high sensitivity and specificity for amyloid protein deposits (18). This feature can be useful for the early diagnosis of CA.

The aim of this study was to assess the diagnostic accuracy of combined ^11^C-PIB positron emission tomography/magnetic resonance (PET/MR) in a cohort of patients with heart failure and suspected cardiac amyloidosis.

## Materials and Methods

### Patients

Twenty-three patients with heart failure and suspected CA diagnosed with echocardiography and electrocardiograph were enrolled in this retrospective study at the First Medical Center of PLA General Hospital between May 1, 2017 and December 31, 2019. Diagnostic criteria included the thickening of the wall of the ventricular septum plus any two of the following criteria: (a) Ultrasound showed characteristic enhanced echo, such as Granular echo, Speckled echo or Ground glass echo; (b) Unexplained low voltage <0.5 mV in the limb leads of the 12-lead electrocardiogram; (c) Left ventricular diastolic function decreased; (d) or Left atrium enlarged. A series of ^11^C-PIB PET/MR, echocardiography, extra-cardiac biopsy, and laboratory tests were performed. Eight healthy volunteers without any signs or symptoms of cardiac disease were also enrolled (5 males and 3 females; age range: 41–65 y). The non-CA patients and healthy subjects were considered as the control group. We compared ^11^C-PIB uptake in the myocardium and the values of several CMR imaging parameters between the patients with CA and control subjects to establish cutoff values. Written informed consent was obtained from all patients prior to recruitment. This study was approved by the Human Ethics Committee of the Chinese PLA General Hospital.

### ^11^C-PIB PET/MR Scanning Parameters

Simultaneous ^11^C-PIB PET and CMR of the heart were performed using a 3T hybrid PET/MR system (Biograph mMR, Siemens Healthineers, Erlangen, Germany). All study subjects were injected with 555 MBq of 11C-PIB through the antecubital vein, and PET data was acquired in list mode using a 20 min table time. The acquisition was started 30 mins after the administration of ^11^C-PIB. PET images were reconstructed with a 256 × 256 matrix with the ordered-subset expectation maximization method (4 iterations, 8 subsets), and post-smoothing was performed using a 4 mm Gaussian filter. PET images of 5 mm slice thicknesses were then displayed along the transversal, coronal, and sagittal planes. Attenuation correction was performed using the respiratory-gated 2-point Dixon sequence, which was acquired before injection of the gadolinium contrast medium. The Dixon imaging parameters were as follows: repetition time (TR) = 3.6 ms; echo time (TE)1 = 1.23 ms; TE2 = 2.46 ms; field-of-view = 500 × 500 mm; and flip angle (FA) = 10°.

Each CMR series was acquired during the expiratory phase with breath holding. The heart was localized by first acquiring two-dimensional (2D) scout images in the transversal, coronal, and sagittal planes. CMR cine images were acquired using ECG-gated 2D-segmented balanced steady-state free precession (bSSFP) sequence. Two-, three-, and four-chamber long-axis and 10–12 short-axis slices covering the LV were acquired to evaluate cardiac motion and function. The key parameters were as follows: TR/TE = 3.3/1.43; FA = 55°–70°; voxel size = 1.6 × 1.6 × 6.0 mm^3^; temporal resolution = 45.6 ms; bandwidth = 962 Hz/pixel. The 5(3)3 and 4(1)3(1)2 MOLLI sequence acquisition schemes were used for native T1 and post-contrast T1 mapping, respectively. Identical images were obtained from the basal, mid, and apical short axis slices of the ventricle and the 4-chamber long-axis slices (19, 20). The parameters were as follows: TR/TE = 2.7/1.12 ms; FA = 35°; voxel size = 1.4 × 1.4 × 8.0 mm^3^. LGE images were generated using a 2D phase-sensitive inversion-recovery (PSIR) gradient-echo pulse sequence with the following parameters: TR/TE = 5.2/1.96 ms FA = 20°; voxel size = 1.4 × 1.4 × 8.03 mm.

### PET/MRI Analysis

PET activity was measured within the LV myocardium by analyzing fused and co-registered PET and LGE-MR images. Myocardial PET uptake was quantified using standard uptake values (SUV) and target-to-background ratio (TBR) after correcting for the blood-pool activity in the descending thoracic aorta. The standard uptake value (SUV) of the myocardium was measured by drawing the contour of the whole LV at an approximate thickness of 10 mm from base to apex. Maximal SUV (SUVmax) was defined as the voxel with the highest uptake among all the volumes of interest (VOIs) analyzed. Mean SUV (SUVmean) was defined as the average SUV of the total voxels in the VOI. The maximal myocardium to blood cavity ratio (TBRmax) was defined as the maximal SUV of the myocardial VOI divided by the mean SUV of the descending thoracic aorta VOI.

CMR functional parameters, native T1, and ECV were measured semi-automatically using a dedicated CMR software, cvi42 version 5.3 (Circle Cardiovascular Imaging, Calgary, Canada) (5). LV ejection fraction (LVEF) and standard parameters of the cardiac structure such as LV mass, ventricle volume, inter-ventricular septum thickness (IVSD), and left ventricular posterior wall thickness (LVPW) were measured by tracing the endocardial and epicardial borders in the long-axis and short axis cine images at the end-systolic and end-diastolic timepoints. T1 values of global LV were obtained by drawing contours around the endocardium and epicardium as well as indicating the inter-ventricular septum on pre-contrast T1 mapping images with indexing for the hematocrit. Native T1 and ECV of global LV were measured by drawing contours around the endocardium and epicardium as well as indicating the inter-ventricular septum on pre-contrast and post-contrast T1 mapping images with indexing for the hematocrit. Global LV native T1 and ECV values were used for further analyses. All ^11^C-PIB PET/MR images were analyzed independently by two experienced investigators in nuclear medicine independently. All disagreements were resolved in consultation with a third investigator.

### Statistical Analysis

The continuous variables are presented as means ± SD and compared between control and CA patient groups using the Mann–Whitney U test. Receiver operating characteristic (ROC) curves were used to determine the diagnostic TBRmax and native T1 values for CA. Spearman's correlation analysis was performed to determine the degree of association (Spearman's r value) between native T1 value and TBRmax. *P* < 0.05 was considered statistically significant. All statistical analyses were performed using the R 4.0.1 Statistical Package (the R foundation for Statistical Computing, Vienna, Austria) and SPSS software v.24.0 (Statistical Package for Social Science; IBM, Chicago, IL, USA).

## Results

### Baseline Characteristics

Among the 23 enrolled patients with heart-failure suspected of having CA, thirteen patients were diagnosed with CA. Ten patients with CA were diagnosed by typical non-invasive detection of cardiac involvement by CMR LGEs and positive Congo-red staining of abdominal fat pad biopsies and bone marrow; 2 patients with CA were diagnosed by diffuse sub-endocardial or transmural LGEs and at least one of the monoclonal protein tests being reported as abnormal (21); 1 patient with CA was diagnosed by a positive abdominal fat pad biopsy, positive genetic test for amyloid, and typical echocardiography patterns, including >12 mm thick left ventricular wall and the appearance of grain scintillation in the myocardial wall. The remaining 10 cases were diagnosed as different types of cardiomyopathy: dilated cardiomyopathy (DCM, *n* = 2), rheumatic heart disease (RHD, *n* = 1), valvular heart disease (VHD, *n* = 1), hypertensive heart disease (HHD, *n* = 3), and hypertrophic cardiomyopathy (HCM, *n* = 3). The baseline characteristics of the enrolled patients are summarized in Table 1.

**Table 1:** Comparison of the baseline clinical data and ^11^C-PIB PET/MR parameters between patients with and without cardiac amyloidosis (CA).

**Patient no**.	**Age**	**Sex**	**Diagnosis**	**ECG low**	**NYHA functional**	**Urine**	**Blood**	**Biopsy**	**Biopsy**	^**11**^ **C-PIB PET/MR**			
				**voltage**	**class**	**IFE**	**IFE**	**(bone marrow)**	**(abdominal wall)**				
										**Visually PET positive**	**TBR**	**T1 native**	**ECV**
1	68	M	CA	+	II	-	NA	NA	+	Yes	3.69	1,525	47
2	67	M	CA	+	IV	+	-	NA	+	Yes	8.08	1,487	52
3	64	M	CA	-	III	+	+	NA	+	Yes	1.77	1,496	50
4	64	F	CA	+	IV	+	+	+	+	Yes	1.30	1,503	51
5	61	F	CA	+	III	+	+	NA	+	Yes	1.82	1,506	61
6	76	F	CA	+	IV	NA	+	NA	-	Yes	5.11	1,432	45
7	51	M	CA	-	III	-	+	+	-	Yes	2.02	1,506	52
8	71	M	CA	+	III	-	-	+	+	Yes	1.21	1,507	65
9	63	M	CA	+	III	NA	-	+	+	Yes	2.44	1,456	51
10	61	M	CA	+	IV	-	-	+	+	Yes	2.24	1,601	54
11	67	F	CA	+	IV	+	-	+	+	Yes	2.75	1,537	56
12	44	F	CA	-	II	-	-	NA	-	No	1.29	1,433	52
13	60	M	CA	-	IV	-	-	NA	+	No	0.92	1,432	38
14	33	M	HHD	-	II	-	-	NA	+	No	0.91	1,419	NA
15	68	M	HHD	-	IV	-	-	NA	+	No	0.94	1,378	32
16	55	M	DCM	-	III	-	-	NA	-	No	0.89	1,315	37
17	70	F	RHD	+	III	-	-	NA	+	No	0.87	1,420	29
18	58	M	VHD	-	IV	NA	-	NA	+	No	0.79	1,349	27
19	73	F	HCM	-	I	-	-	NA	+	No	0.89	1,402	34
20	28	M	HCM	-	II	-	NA	NA	+	No	0.78	1,427	33
21	32	F	HCM	-	II	-	-	+	-	No	0.82	1,363	31
22	67	F	DCM	-	III	-	-	NA	-	No	0.91	1,363	40
23	56	F	HHD	+	III	-	-	+	-	No	0.94	1,467	NA

*ECG, electrocardiograph; NYHA, new york heart association; IFE, immunofixation electrophoresis; PET, positron emission tomography; TBR, tissue-to-blood background ratio; ECV, extracellular volume; M, male; F, female; HHD, hypertensive heart disease; DCM, dilated cardiomyopathy; RHD, rheumatic heart disease; VHD, valvular heart disease; HCM, hypertrophic cardiomyopathy*.

### Comparison of Clinical and Biochemical Biomarkers Between CA and Non-CA Patients

We did not observe any significant differences in age between CA patients, non-CA patients, and healthy subjects (*P* > 0.05). The BMI values of non-CA patients were significantly higher compared with the CA patients and healthy subjects (*P* = 0.033). We also did not observe any significant differences in the serum cardiac troponin-I (cTnI), calcium, and creatinine values between CA patients and non-CA patients (*P* > 0.05). CA patients showed significantly higher levels of NT-proBNP (13011.46 ± 11726.99 pg/mL vs. 4709.30 ± 5428.82 pg/mL, *P* = 0.036) and blood-free light chain kappa/lambda (1.88 ± 5.77 vs. 0.85 ± 0.31 mg/dL, *P* = 0.021) compared with the non-CA patients. The 13 CA patients were classified under NYHA classification I (*n* = 0), II (*n* = 2), III (*n* = 5), and IV (*n* = 6), respectively (Table 2).

**Table 2:** Baseline clinical characteristics of patients with cardiac amyloidosis (CA), those without CA (non-CA), and healthy control subjects.

**Characteristics**	**CA (n:13)**	**Non-CA (n:10)**	**Controls (n:8)**	***P***
**General parameters**	
Age (years)	62.9 ± 8.2	54.0 ± 17.0	47.9 ± 13.8	0.105
Female/Male	5/8	5/5	3/5	
BMI (kg/m^2^)	22.30 ± 3.04	26.52 ± 4.28	23.84 ± 2.62	0.033
HTN/CHD/DM/Arrhythmia/AF	11/3/1/5/6	7/2/4/4/1	-	-
**Biomarkers**	
cTnI (ng/ml)	0.13 ± 0.14	0.07 ± 0.07	-	0.166*
NT-proBNP (pg/ml)	13011.46 ± 11726.99	4709.30 ± 5428.82	-	0.036*
Calcium (mg/ml)	2.17 ± 0.11	2.16 ± 0.15	-	0.879*
Creatinine (mg/ml)	117.16 ± 78.90	129.79 ± 70.43	-	0.605*
Blood free light chain Kap/Lam (mg/dl)	1.88 ± 5.77	0.85 ± 0.31	-	0.021*
**CMR parameters**	
LVEF (%)	48.1 ± 9.4	42.2 ± 15.1	65.2 ± 1.8	<0.001
LV mass (g)	164.3 ± 42.0	181.9 ± 70.1	94.2 ± 22.8	0.002
LVEDV (ml)	101.6 ± 28.2	180.8 ± 67.7	94.0 ± 16.7	0.001
LVESV (ml)	53.9 ± 22.9	108.8 ± 67.4	32.8 ± 8.7	<0.001
IVSD (cm)	15.0 ± 1.6	12.0 ± 2.9	9.0 ± 1.8	<0.001
LVPW (cm)	10.3 ± 2.2	9.8 ± 3.0	7.9 ± 0.9	0.030
Native T1 value (ms)	1493.9 ± 48.1	1390.3 ± 44.8	1264.6 ± 25.6	<0.001
ECV	51.9 ± 6.7	32.5 ± 3.8	-	<0.001
TBR	2.66 ± 1.99	0.85 ± 0.06	0.88 ± 0.07	<0.001

*BMI, body mass index; HTN, hypertension; CHD, coronary heart disease; DM, diabetic mellitus; AF, atrial fibrillation; cTnI, cardiac troponin; NT-proBNP, N-terminal pro-B-type natriuretic peptide; CMR, cardiac magnetic resonance; LVEF, left ventricular ejection fraction; LVEDV, left ventricle end-diastolic volume; LVESV, left ventricle end-systolic volume; IVSD, interventricular septal thickness at diastole; LVPW, left ventricular posterior wall thickness; ECV, extracellular volume; TBR, maximum target-to-background ratio*.

The symbol * represents the comparison between the two groups.

### The Echocardiography Diagnositic Parameters and PET/MR Structural and Functional Parameters

**Figure 1:**
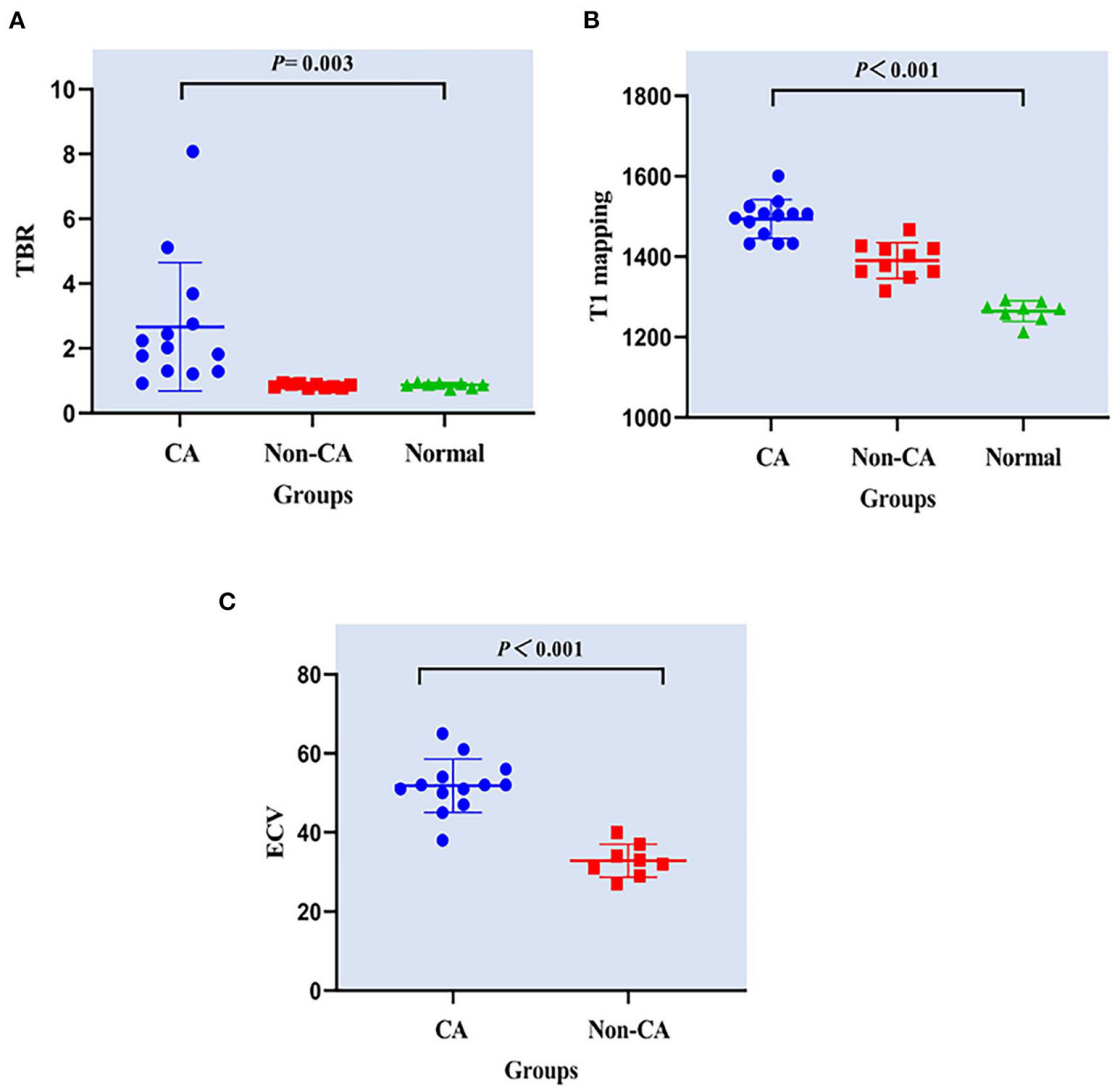
Comparison of **(A)** TBR, **(B)** T1 mapping, and **(C)** ECV values between CA patients, non-CA patients, and healthy control subjects. CA, cardiac amyloidosis; TBR, maximum target-to-background ratio; ECV, extracellular volume. **(A)** PET data showed that the TBR was significantly higher in the CA patients compared with the non-CA patients and healthy subjects (2.66 ± 1.99 vs. 0.85 ± 0.06 vs. 0.88 ± 0.07; *P* < 0.05). **(B)** The native T1 values were significantly higher in the CA patients compared with the non-CA and healthy subjects (1493.9 ± 48.1 vs. 1390.3 ± 44.8 vs. 1264.6 ± 25.6; *P* < 0.05). **(C)** The ECV values were significantly higher for the CA patients compared with the non-CA patients (51.9 ± 6.7 vs. 32.9 ± 4.2; *P* < 0.05).

The echocardiography data for CA patients and non-CA patients are summarized in Table 3. There were no differences in the echocardiographic parameters between the CA patients and the non-CA patients, except for a significant difference in the LV end-diastolic volume (83.2 ± 28.4 vs. 134.4 ± 44.0 ml, *P* < 0.05). We observed significant differences among several CMR parameters (LVEF, IVSD, LVmass, LVEDV, LVESV, LVPW, native T1 value, and ECV) and TBR between CA patients, non-CA patients, and healthy subjects (*P* < 0.05; Table 2). PET data showed that the maximal myocardial ^11^C-PiB uptake was significantly higher in the CA patients compared with the non-CA patients and healthy subjects (0.88 ± 0.07) (2.66 ± 1.99 vs. 0.85 ± 0.06 vs. 0.88 ± 0.07; *P* < 0.05; Figure 1A). The native T1 values were significantly higher in the CA patients compared with the non-CA and healthy subjects (1493.9 ± 48.1 vs. 1390.3 ± 44.8 vs. 1264.6 ± 25.6; *P* < 0.05; Figure 1B). The ECV values were significantly higher for the CA patients compared with the non-CA patients (51.9 ± 6.7 vs. 32.9 ± 4.2; *P* < 0.05; Figure 1C).

**Table 3:** Echocardiography data from CA patients and non-CA patients.

	**Clinical diagnositic of cardiac amyloidosis**		

	**Yes (*****n** **=*** **13)**	**No (*****n** **=*** **10)**	***P***
Ventricular septum wall thickness, mm	15.0 ± 2.4	13.4 ± 2.0	0.103
LV ejection fraction, %	47.0 ± 11.8	47.8 ± 14.9	0.887
LV end-diastolic volume, ml	83.2 ± 28.4	134.4 ± 44.0	0.003
Decrease of LV	9	7	-
Diastolic function
Enlargement of LA	12	7	-
Granular echo	11	6	-
Speckled echo	1	-	-
Ground glass echo	1	-	-

*LVEF, left ventricular ejection fraction*.

**Figure 2:**
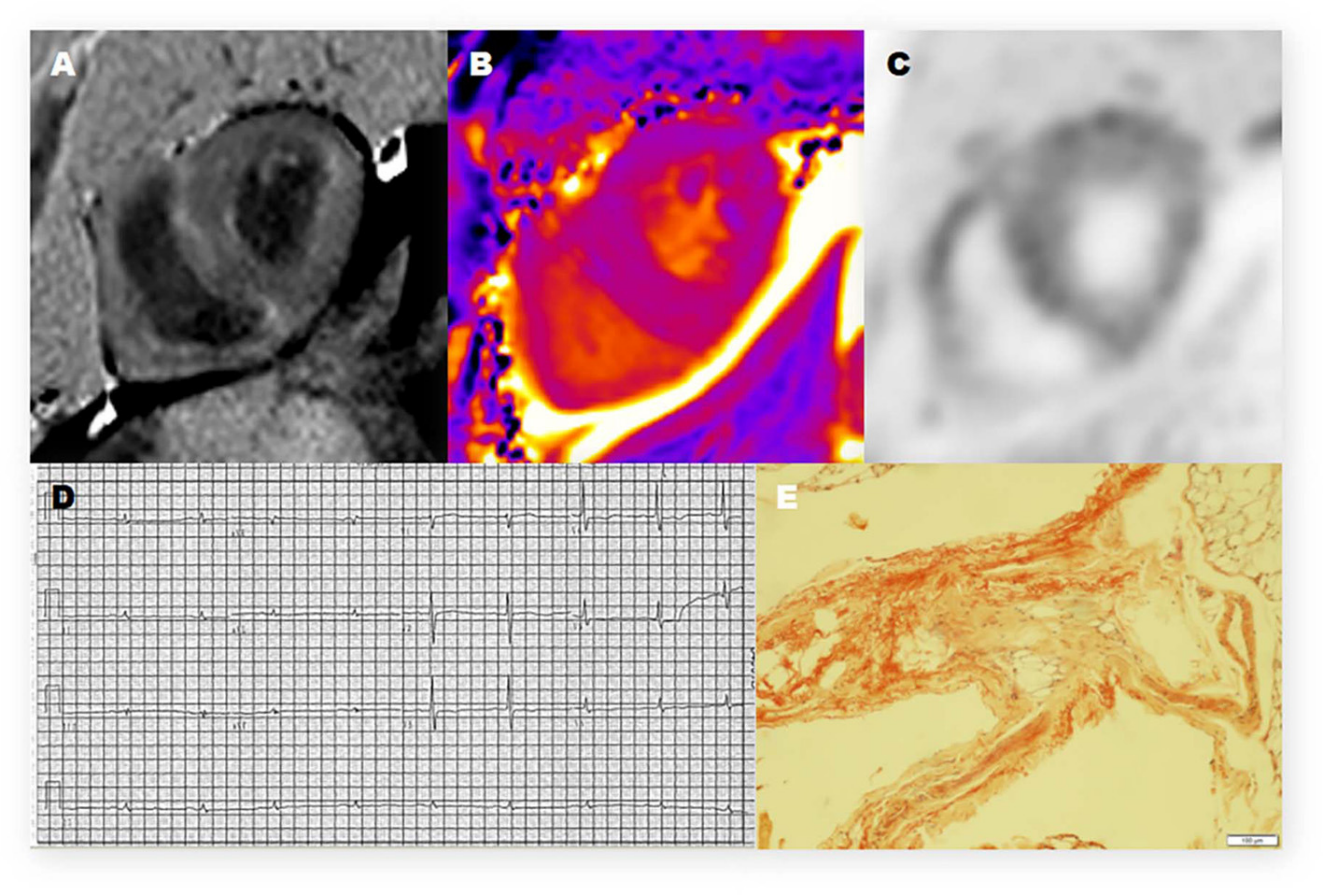
Representative ^11^C-PiB PET/MR, electrocardiogram, and histologic photomicrograph of one CA patient. **(A)** Late-gadolinium enhancement (LGE)-CMR image demonstrates a diffuse transmural enhancement pattern. **(B)** Native T1 mapping shows that the myocardial T1 value was 1,569 ms. **(C)** The PET/MR image of the myocardium shows strong uptake of ^11^C-PIB in the left ventricle (LV) and right ventricle (RV). **(D)** The electrocardiogram (12-lead) shows low voltage in the limb leads. **(E)** Histopathologic examination shows positive Congo red staining and amyloid deposits in the adipose tissue.

Twelve of the thirteen CA patients showed characteristic LGE patterns including diffuse transmural myocardial enhancement, typical subendocardial ring enhancement, and heterogeneous transmural LGE pattern. Only one patient did not show any characteristic LGE pattern. Gadolinium-enhanced CMR was not performed in 2 non-CA patients because of significant azotemia (creatinine clearance < 30 ml/kg/min). In the remaining 8 non-CA patients, we observed patchy LGE enhancement patterns including discrete areas of LGE or diffuse areas of LGE in less than half of the short axis images (Table 4). LGE patterns, T1 mapping, and PET images of a representative CA patient are shown in Figure 2. Visual inspection of PET images demonstrated positive ^11^C-PIB uptake in 12 out of 13 CA patients. However, none of the non-CA patients and healthy subjects showed any visible ^11^C-PIB uptake.

**Table 4:** Baseline late gadolinium enhancement (LGE) patterns for all cardiac amyloidosis (CA) and non-CA patients based on ^11^C-PIB PET/MR data.

**Patients**	**Diagnosis**	^**11**^ **C-PIB PET/MR**	
		**Uptake**	**LGE pattern**
1	CA	Yes	Diffuse transmural myocardial enhancement
2	CA	Yes	Diffuse transmural myocardial enhancement
3	CA	Yes	Diffuse transmural myocardial enhancement
4	CA	Yes	Subendocardial ring enhancement
5	CA	Yes	Diffuse transmural myocardial enhancement
6	CA	Yes	Diffuse transmural myocardial enhancement
7	CA	Yes	Diffuse transmural myocardial enhancement
8	CA	Yes	Subendocardial ring enhancement
9	CA	Yes	Heterogeneous transmural enhancement
10	CA	Yes	Subendocardial ring enhancement
11	CA	Yes	Subendocardial ring enhancement
12	CA	No	Diffuse transmural myocardial enhancement
13	CA	No	Negative
14	HHD	No	Not done because of azotemia
15	HHD	No	Left ventricular apex subendocardium enhancement
16	DCM	No	Enhancement of the left ventricular septal middle layer
17	RHD	No	Negative
18	VHD	No	Enhancement of the left ventricular septal middle layer
19	HCM	No	Enhancement of the junction between interventricular septum and inferior wall
20	HCM	No	Negative
21	HCM	No	Enhancement of the medial anterior wall and inferior wall papillary muscle
22	DCM	No	Enhancement of apex, anterior wall papillary muscle, interwall and inferior wall subendocardium
23	HHD	No	Not done because of azotemia

*LGE, late gadolinium enhanced*.

ROC curve analysis using a TBR cutoff value of 1.09 showed that the area under the curve (AUC) value for discriminating CA patients from the controls (10 non-CA patients and 8 healthy subjects) was 0.99 (95% CI: 0.96–1.00) with a sensitivity of 92% (95% CI: 62–100%) and specificity of 100% (95% CI: 78–100%) (Table 4). The positive predictive value (PPV) using a TBR cutoff value of 1.09 was 100% (95% CI: 70–100%) and the negative predictive value (NPV) was 95% (95% CI: 72–100%). The PPV, AUC, sensitivity, and specificity values for both ECV and TBR were similar at the ECV cutoff value of 42.5, but NPV [89% (95% CI: 51%–99%)] was lower than TBR. The AUC value for discriminating CA patients from controls using a T1 mapping cutoff value of 1,429.5 was 0.98 (95% CI: 0.96–1.00) with a sensitivity of 100% (95% CI: 72–100%) and specificity of 94% (95% CI: 71–100%). The positive predictive value with this cutoff was 93% (95% CI: 64–100%) and the negative predictive value was 100% (95% CI: 77–98%). The other CMR parameters are shown in Table 5.

**Table 5:** Differential diagnostic efficiency of TBR and CMR parameters between cardiac amyloidosis (CA) patients and control subjects (non-CA patients‘ and healthy subjects).

	**Cutoff**	**Sensitivity (%)**	**95% CI (%)**	**Specificity (%)**	**95% CI (%)**	**AUC**	**95% CI (%)**
TBR	1.09	92	62–100	100	78–100	0.99	0.96–1.00
T1 mapping value	1,429.5	100	72–100	94	71–100	0.98	0.95–1.00
ECV	42.5	92	62–100	100	78–100	0.99	0.96–1.00
IVSD (cm)	13.4	100	71–100	83	57–96	0.91	0.79–1.00
LVPW (cm)	8.9	85	54–97	67	41–86	0.71	0.52–0.90
LVEF ()	51.4	77	46–94	72	46–89	0.66	0.46–0.86
LVESV (ml)	64.2	85	54–97	39	18–64	0.53	0.32–0.74
LVEDV (ml)	104.1	77	46–94	72	46–89	0.70	0.51–0.89
LV mass (g)	128.4	85	54–97	67	41–86	0.67	0.47–0.87

*AUC, area under the curve; CI, confidence interval; TBR, maximum target-to-background ratio; ECV, extracellular volume; IVSD, interventricular septal thickness at diastole; LVPW, left ventricular posterior wall thickness; LVEF, left ventricular ejection fraction; LVESV, left ventricle end-systolic volume; LVEDV, Left ventricle end-diastolic volume*.

**Figure 3:**
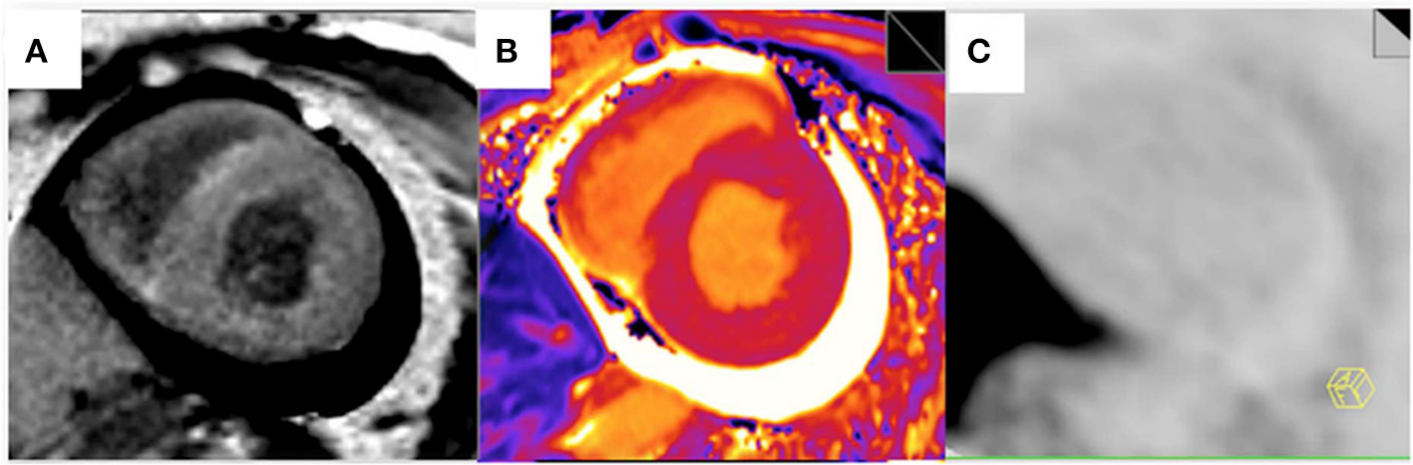
Myocardial LGE, T1 mapping, and PET images from the false-negative CA patient. **(A)** A late gadolinium enhancement image shows a diffuse transmural delayed enhancement pattern in the myocardium. **(B)** The native T1 value was 1,401 ms. **(C)** 11C-PiB PET staining of the myocardium was negative.

**Figure 4:**
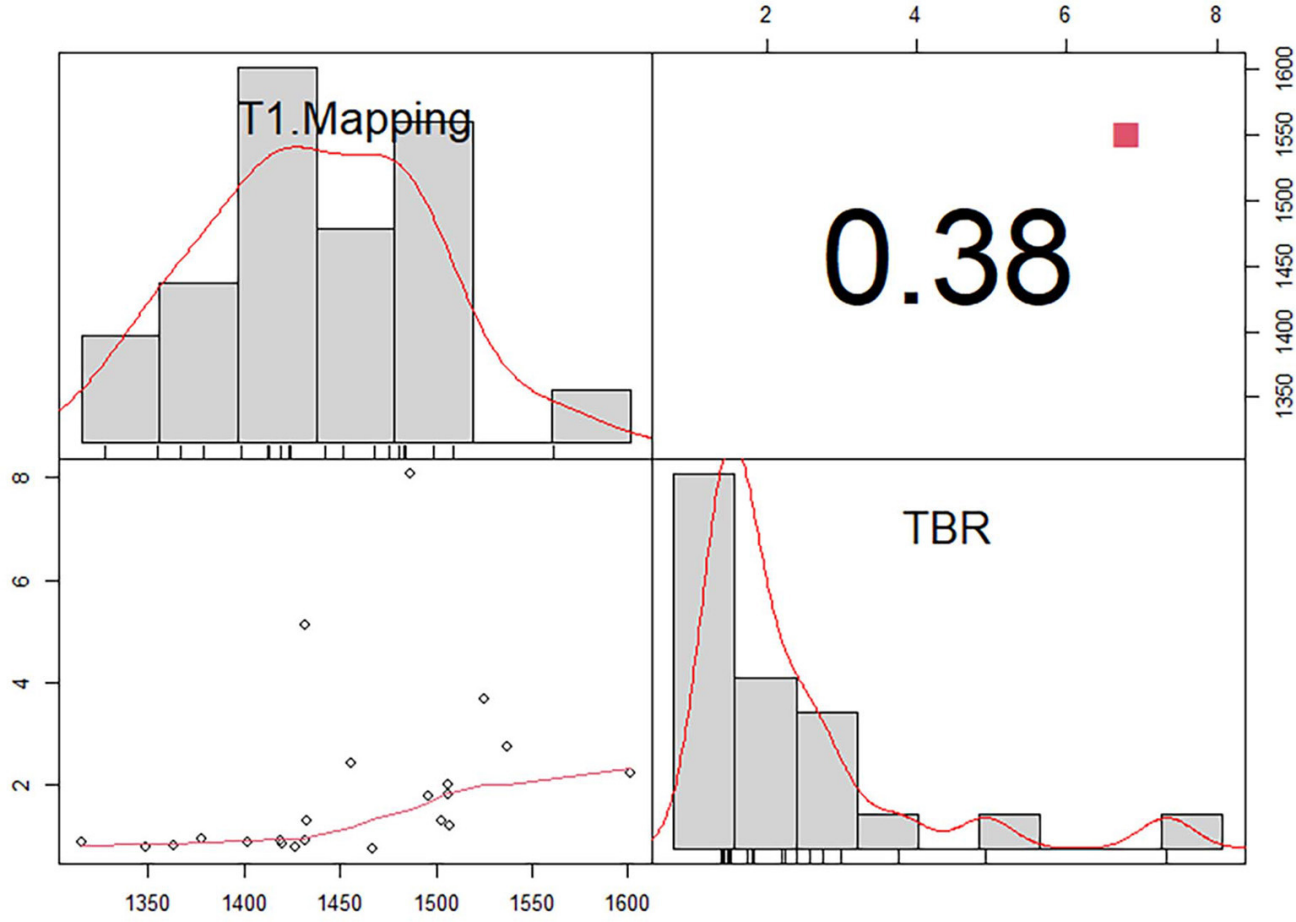
Correlation between T1 mapping and maximum target-to-background ratio (TBR) in patients with (CA) and without cardiac amyloidosis (non-CA).There is a positive correlation between native T1 mapping value and TBR in CA and non-CA patients (*r* = 0.38, *P* = 0.0004).

False-positive ^11^C-PIB PET/MR scan results were not observed in any of the non-CA patients and healthy subjects using a TBR cut-off value of 1.09. In contrast, one CA patient (Patient #13) showed a false-negative ^11^C-PIB PET/MR result (maximal LV myocardium to blood cavity ratio of 0.92) (Figure 3). We observed positive ECV and native T1 mapping values in a PET-negative CA patient who did not show a typical CMR LGE pattern for cardiac amyloidosis, thereby prompting false-positive diagnosis of CA. We did not observe any false positive cases in the non-CA and healthy control groups with a ECV cut-off value of 42.5, but the CA patient with false-negative ^11^C-PIB (Patient #13) also showed false-negative ECV result. None of the CA patients were false-negative and none of the healthy subjects were false-positive when the cut-off value for native T1 mapping was 1,429.5 ms However, one non-CA patient showed false-positive result because the native T1 mapping value was 1467 ms. The ^11^C-PIB PET result of this patient was negative, and MR LGE was not performed on this patient because of renal insufficiency. A positive correlation between native T1 mapping value and TBR in CA and non-CA patients was also observed (*r* = 0.38, *P* = 0.0004, Figure 4).

## Discussion

Our study demonstrated that ^11^C-PIB PET/MR was a highly sensitive and accurate method to confirm cardiac amyloidosis. Moreover, we demonstrated that a combination of TBRmax values derived from ^11^C-PIB PET scans, T1 mapping values, and ECV values derived from CMR can be used for accurate diagnosis of CA.

CA is caused by extracellular and intracellular accumulation of insoluble and misfolded fibrillar amyloid protein and shows clinical features resembling restrictive cardiomyopathy (2). Endomyocardial biopsy, the gold standard for diagnosis of CA, is invasive and cannot be performed routinely (22). Moreover, focal myocardial biopsy does not provide information regarding the overall myocardial amyloid load and active accumulation of the amyloid protein. Therefore, there is an urgent need for techniques that can accurately and non-invasively confirm cardiac amyloidosis in clinical settings. Previous studies (23, 24) have demonstrated that ^11^C-PIB PET/CT is a valuable tool for the non-invasive diagnosis of CA with ≥ 95% sensitivity and specificity (18). Our study showed that ^11^C-PIB PET distinguishes CA patients from both non-CA patients and healthy controls with 94% sensitivity and 100% specificity. Moreover, 11C-PIB-PET was 99% accurate in the positive diagnosis of CA. Rosengren et al. demonstrated that 11C-PIB-PET detected CA in different amyloid subtypes with 89 to 100% accuracy in a dual-center setting (25). Therefore, our results are consistent with these previous findings and suggest that 11C-PIB PET is an independent diagnostic indicator of CA.

LGE-CMR has shown great promise in clinical diagnosis and prognosis because it offers high spatial resolution and the ability to identify pathology in the extracellular space (26). CA patients show diverse patterns of late gadolinium enhancement, such as global subendocardial enhancement, transmural LGE, and patchy focal LGE. In some CA cases, it is very difficult to quantify the degree of abnormality based on LGE-CMR. A recent meta-analysis of seven published studies showed that the accuracy of CMR-based LGE in the positive diagnosis of CA was high with 85% sensitivity and 92% specificity. In our study, 12 out of 13 CA patients showed global left ventricular LGE, a typical enhancement pattern of cardiac amyloidosis. In non-CA patients, we did not observe any typical CA enhancement. However, some patients showed patchy enhancement characteristics, which were indistinguishable from non-CA patients.

The native T1 value has been proposed as a quantitative parameter for evaluating CA with diffuse disease. Furthermore, native T1 mapping eliminates the need for gadolinium and can be used as an alternative method for assessing CA patients with renal insufficiency (27). ECV is more reproducible than absolute T1 mapping values. Moreover, ECV is more advantageous than LGE because it can quantify expansion of the extracellular space based on the region of interest drawn on the ECV maps. Our study showed that the diagnostic accuracy of native T1 values and ECV were significantly higher than the other CMR parameters, with the AUC value of ECV being the highest. However, at a molecular level, both native T1 mapping and ECV are not specific to amyloidosis (6, 28). In contrast, ^11^C-PIB PET is a useful method for direct evaluation of overall myocardial amyloid load in cardiac amyloidosis. In our study, the diagnostic accuracy of both TBR and ECV was 99%. Native T1 showed higher sensitivity (100 vs. 94%) and lower specificity (94 vs. 100%) than TBR and ECV. In our study, one CA patient (Patient #13) was false-negative for both ^11^C-PIB PET and ECV. The CMR result of this patient did not show any LV LGE, but the nativeT1 mapping results were positive. Therefore, patient #13 was positively diagnosed as CA based on the native T1 mapping data. The cases of patients #14 and #23 demonstrated unique superiority of ^11^C-PIB PET for differential diagnosis of cardiac amyloidosis from other cardiomyopathies in the setting of significant azotemia, a clinical condition that prevents contrast-enhanced CMR from being performed. Although the native T1 mapping results were positive for patient #23, the PET results were negative. Therefore, patient #23 was classified as non-CA based on the PET results.

We also analyzed the correlations between TBR values derived from ^11^C-PIB PET and native T1. Our analysis showed that TBR and native T1 in PET/MR can be used to diagnose CA patients that cannot undergo delayed enhancement because of renal insufficiency. Hence, the combination of the 2 diagnostic indices played a complementary role and improved the diagnostic accuracy of CA. We did not obtain ECV values for some patients who did not undergo delayed enhancement. Both ECV and TBR showed the same AUC (0.99), sensitivity (92%), specificity (100%) and PPV (100%). However, ECV showed lower NPR compared with TBR (89 vs. 95%). This suggested that ECV can be used as an independent diagnostic index for CA.

There are several limitations to this study. Firstly, the sample size of study subjects was small. Therefore, our results need to be confirmed in large cohort studies. Secondly, we could not complete typing of myocardial amyloidosis in this study because (1) endomyocardial biopsies were not performed for some patients; (2) bone marrow biopsies were performed for only a few patients; and (3) laboratory tests for urine and blood IFE were not performed for all patients. Fontana et al. (29) showed that native T1 values were relatively higher, and ECV values were lower patients with immunoglobulin light chain amyloidosis compared with those with transthyretin amyloidosis (ATTR). Ezawa and Katoh (24) showed that ^11^C-PIB uptake was lower in patients with ATTR compared with the patients with AL. Therefore, the diagnostic value of ^11^C-PIB PET/MR imaging for CA requires further evaluation according to the cardiac amyloidosis type because the underlying mechanisms are not completely known. Finally, normal ranges vary for different CMR systems and T1 mapping sequences (30). Normal T1 values are higher when measured at 3T with different sequences and typically with newer versions of mapping compared with older ones. This is a significant obstacle to using native T1 mapping in clinical practice. Therefore, uniform guidelines and normal ranges need to be established for different CMR techniques and sequences so that data can be compared between different patients that have undergone MRI scans at different facilities.

Although endomyocardial biopsy is the golden standard for diagnosis of CA, extrapolating the amyloid content in line with the biopsy sample to the entire heart may be inaccurate, especially in the early CA when the amyloid deposits may not be extensive or diffuse (31). Quantification of amyloid burden is currently based on assessment of wall thickness, LV mass, ECV, or semi-quantitative index on amyloid PET imaging (32). ^11^C-PIB PET/MR offers substantial advantages. It is non-invasive, quantitative, fuses quantitative parameters of PET and MR to estimate whole-heart cardiac amyloid burden, and can be easily repeated to monitor response to therapy. For patients clinically suspected of CA with heart failure ^11^C-PIB PET/MR is valuable in CA patients with contraindications to gadolinium (due to renal dysfunction). Our study confirms that ^11^C-PIB PET/MR multi-parameter imaging provides additional evidence for the diagnosis of CA in the absence of LGE. Moreover, the high sensitivity and specificity of ^11^C-PIB PET/MR make it possible to detect CA early.

## Conclusions

In conclusion, this study showed that ^11^C-PIB PET/MR accurately diagnosed cardiac amyloidosis with high sensitivity and specificity. ^11^C-PIB PET/MR showed structural and functional changes in CA patients and helped to accurately determine the location and extent of amyloid protein deposition. Our study also demonstrated the advantages of using multiple parameters and multi-sequence imaging characteristics of CMR in combination with the high specificity of ^11^C-PIB PET for the accurate diagnosis of CA.

## Data Availability Statement

The raw data supporting the conclusions of this article will be made available by the authors, without undue reservation.

## Ethics Statement

The studies involving human participants were reviewed and approved by the Human Ethics Committee of the Chinese PLA General Hospital. The patients/participants provided their written informed consent to participate in this study. Written informed consent was obtained from the individual(s) for the publication of any potentially identifiable images or data included in this article.

## Author Contributions

XB, JL, WD, JA, RW, BX, and ZG: conception and design. XB, JL, XZ, and WD: data collation. XB, JL, and GW: statistical Analysis. XB and JL: article writing. GW, JA, WD, BX, and ZG: article revision. All authors contributed to the article and approved the submitted version.

## Funding

Granting agencies: National Clinical Research Center for Geriatric Diseases, Chinese PLA General Hospital. Grant number: NCRCG-PLAGH-2019012.

## Conflict of Interest

JA is employed at Siemens Healthcare Ltd. The remaining authors declare that the research was conducted in the absence of any commercial or financial relationships that could be construed as a potential conflict of interest.

## Publisher's Note

All claims expressed in this article are solely those of the authors and do not necessarily represent those of their affiliated organizations, or those of the publisher, the editors and the reviewers. Any product that may be evaluated in this article, or claim that may be made by its manufacturer, is not guaranteed or endorsed by the publisher.
